# Cæsarian Section

**Published:** 1868-03

**Authors:** 


*Cæsarian Section.*—Prof. Wm. Warren Green reports in the *Boston Medical and Surgical Journal* for February, a successful case, in which mother and child were saved. The details of the case are interesting, and if possible, we shall republish it in our next journal.

